# The newer Opioid Agonist Treatment with lower substitutive opiate doses is associated with better toxicology outcome than the older Harm Reduction Treatment

**DOI:** 10.1186/s12991-016-0109-z

**Published:** 2016-11-25

**Authors:** Jacopo V. Bizzarri, Valentina Casetti, Livia Sanna, Angelo Giovanni Icro Maremmani, Luca Rovai, Silvia Bacciardi, Daria Piacentino, Andreas Conca, Icro Maremmani

**Affiliations:** 1 Department of Psychiatry of Bolzano, Bolzano, Italy; 2 Vincent P. Dole Dual Diagnosis Unit, Department of Neurosciences, Santa Chiara University Hospital, University of Pisa, Via Roma, 67 56100 Pisa, Italy; 3 AU-CNS-Association for the Application of Neuroscientific Knowledge to Social Aims, Pietrasanta, Italy; 4 G De Lisio Institute of Behavioural Sciences, Pisa, Italy

**Keywords:** Harm reduction treatment, Agonist opioid treatment, Urinalyses, Polysubstance use, Heroin, Cannabinoids, Cocaine

## Abstract

**Background:**

Charge-free heroin use disorder treatment in Italy follows two main approaches, i.e., harm reduction treatment (HRT) strategy in community low-threshold facilities for drug addiction and opioid agonist treatment (OAT) in high-threshold facilities for opioid addiction, focusing on pharmacological maintenance according to the Dole and Nyswander strategy. We aimed to compare the impact of HRT and OAT on patient outcome, as assessed through negativity for drugs on about 1-year urinalyses.

**Methods:**

We examined retrospectively the urinalyses of HRT and OAT patients for which at least four randomly sampled urinalyses per month were available for about 1 year, during which patients were undergoing methadone or buprenorphine maintenance; urinalyses focused on heroin, cocaine, cannabinoids, and their metabolites.

**Results:**

Included were 189 HRT and 58 OAT patients. The latter were observed for a significantly longer period. There was a higher proportion of heroin- and cocaine-clean urinalyses in OAT patients, with cocaine-clean urinalyses discriminating best between the two groups. OAT patients were older, with longer dependence duration, more severe addiction history, and received lower methadone doses. Buprenorphine maintenance was more often associated with heroin-clean urinalyses. The higher the methadone doses, the lower were the percentage of heroin-clean urinalyses in HRT patients (negative correlation).

**Conclusions:**

The OAT approach was related to higher recovery and polyabuse abstinence rates compared to the HRT approach, despite greater severity of substance use, psychiatric and physical comorbidities. Our results are consistent with the possibility to use lower maintenance opiate doses (after induction and stabilization in methadone treatment according to Dole and Nyswander methodology) in treating heroin addiction. This seemed to be impossible adopting the currently accepted HRT model.

## Background

Opioid misuse is a long-term and often chronic condition [1] that has an estimated prevalence of 0.40 % in the whole world, 0.82 % in Europe, and 0.43 in Italy [2, 3]. It is associated with severe consequences for both individuals and society [4]. Heroin use is associated with somatic complications, including hepatitis B, hepatitis C, and immunodeficiency virus (HIV) infections, as well as pulmonary and cardiovascular complications [5], resulting in an increased risk of mortality [6]. Individuals with opioid use disorder also have a higher prevalence of psychiatric disorders, particularly depression, anxiety, and personality disorders [7]. Moreover, opioid use disorder results in an economic burden for society, in terms of medical expenses, social welfare, loss of productivity, and costs of criminal activities [8].

The main pharmacological approach in treating opioid dependence involves opioid agonist maintenance [4, 9]. It consists in the replacement of the illegal drug, which has a 2- to 3-h half-life, with a prescribed opioid, which has a longer half-life and a good μ-receptor activity [10]. This approach aims to give relief from drug craving and withdrawal symptoms without providing patients with an intense high [9, 10]. The medications most frequently used are methadone and buprenorphine [9], which have proven to be effective in reducing heroin use, maintaining prolonged periods of abstinence, increasing retention in drug treatment, and reducing the risk of overdose-related death [4, 11, 12]. Given their proven efficacy, these two drugs have been included by the World Health Organization in the Model List of Essential Medicines [4].

Charge-free heroin use disorder treatment in Italy follows two main approaches, i.e., harm reduction treatment (HRT) strategy [13, 14] in community low-threshold facilities for drug addiction and opioid agonist treatment (OAT) in high-threshold facilities for opioid addiction, focusing on pharmacological maintenance according to the Dole and Nyswander strategy [9, 15–17]. Traditionally, physicians consider complete abstinence as the best and only treatment for substance use. However, in recent years this paradigm has been losing ground. The alternative treatment called HRT has been gaining stronger empirical support and wider practice, even if, as all evidence-based approaches [18], it is in need of further development. The rationale of HRT is that patients will continue to use illicit drugs to some degree, as in some of them it is just unavoidable, and rather than pushing them into an abstinence they will not immediately or fully accept, physicians will step into provide support that can make drug use less harmful. It has been demonstrated that methadone-based HRT, in which the use of illicit drugs is tolerated, is strongly related to decreased mortality from natural causes and from overdoses [19]. In HRT, doses and treatment duration are usually limited, regardless of clinical indication [20, 21], suggesting the possible usefulness of increasing them [22–25]. Patients are allowed to negotiate dosage lowering, regardless of urinalyses results, and to have their medication tapered earlier than advisable on the basis of the scientific literature. A weakness of HRT is that physicians do not seem eager to work with patients who continue to use drugs, even if in a safer or more controlled manner, but without achieving a long-term withdrawal. A study carried out in Canada [26] found that only 56 % of physicians working in addiction treatment facilities would be willing to provide long-term replacement therapy. OAT with methadone or buprenorphine has long been established as the gold standard in treating opioid use disorders, although not without fairly substantial financial and personal costs for patients taking part in this treatment [27]. OAT has been shown to reduce the effects of opioid withdrawal and cravings, increase retention in treatment, and reduce risk behaviors that lead to the transmission of HIV and viral hepatitis [28–31]. However, abstinence as a medium- and long-term goal is not always achieved and some patients continue to abuse heroin during treatment [32]. Moreover, they frequently abuse other non-opiate drugs, such as cocaine and cannabinoids [8, 33]. Concurrent cocaine use is particularly challenging in clinical practice, as it is associated with poor prognosis both in terms of treatment drop-out and heavy concurrent use of heroin [33–37]. There is also an ongoing debate regarding the qualitative characteristics that define the optimal OAT intervention, namely the treatment threshold. “Treatment thresholds” are defined as barriers patients may face prior to and during treatment. There are increasing numbers of studies suggesting better treatment outcomes in low-threshold designs compared to high-threshold ones, due to accessibility so as to avoid waiting lists, use of personalized treatment options regarding medication choice and dose titration, and a treatment design that focuses on maintenance with emphasis on the retention of low-adherence patients [38]. In OAT four phases are envisaged [4, 9]: during the induction phase street heroin or other opioid drugs are substituted with an opioid agonist. During the stabilization phase physicians determine the appropriate agonist dose for long-term maintenance. The doses are gradually increased, reflecting the results of urinalyses, until they produce opioid blockade and patients become tolerant to street heroin. Once this requirement is fulfilled, patients are defined as “stabilized” and the dose at which this goal has been accomplished is referred to as the “stabilization dose”. No upper limit for doses exists. However, a time limit exists: patients who cannot achieve stabilization within 1 year are transferred to the HRT approach. Patients are not allowed to raise or lower doses by themselves. Take-home doses, without limitations and for maximum a week, are allowed once full compliance with the rules of the program is reached. This is the maintenance phase. During the medically supervised withdrawal phase a slow reduction of the agonist dose is applied until patients are completely drug-free. Psychosocial support is usually provided [9], as it has been demonstrated that the association of pharmacotherapy and psychosocial interventions improves results at follow-up [13, 39].

Due to uncertainty regarding which treatment is more effective in the medium and long term, our aim was to compare the impact of HRT and OAT on patient outcome, as assessed through negativity for drugs on about 1-year regularly performed urinalyses. We hypothesized that OAT patients would show better outcomes in terms of heroin, cocaine, and cannabinoids use than HRT ones during maintenance and supervised withdrawal phases. We did not expect to find a relationship between this better outcome and the agonist opioid dose taken by patients.

## Methods

### Design of the study

We conducted a retrospective and naturalistic study to compare urinalyses results of HRT and OAT patients for which at least four randomly sampled urinalyses per month were available for about 1 year, during which patients were undergoing methadone or buprenorphine maintenance; urinalyses focused on heroin, cocaine, cannabinoids, and their metabolites. OAT patients were monitored after having completed their induction and stabilization phase. Given that maintenance phase in OAT patients did not generally start before 6 months of treatment, monitoring of HRT patients did not start before 6 months of treatment.

### Sample

We took into account a dataset of more than 1000 heroin-dependent patients, who had requested treatment during the period 1994–2015 at the Dual Diagnosis Unit of Santa Chiara University Hospital in Pisa, Italy, and at the Drug Addiction Service of Bolzano, Italy. All patients received a diagnosis of opioid dependence with physical dependence according to DSM III/III-R/IV/IV-R/5 criteria and gave their informed consent to the anonymous use of their clinical data for non-profit research.

Patients were selected if urinalyses for heroin, cocaine, and cannabinoids had been monitored for almost a year and collected randomly at least four times per month. As a result, 247 patients were included in the study: 189 HRT patients and 58 OAT patients.

### Instruments

Information regarding patients’ addiction history was obtained from the drug addiction history questionnaire (DAH-Q) [40]. As for toxicological urinalyses, urine specimens were tested for opioids, cocaine, cannabinoids, and their metabolites.

#### Drug addiction history questionnaire (DAH-Q)

The DAH-Q [40] is a multi-dimensional questionnaire that gathers addiction-related information and is administered by a psychiatrist. It comprises the following eight areas: (1) demographic data; (2) physical health (hepatic, vascular, and lymphatic pathology, gastrointestinal disorders, sexual disorders, dental pathology, HIV seropositivity); (3) mental health (awareness of illness, memory disorders, mood disorders, anxiety disorders, thought disorders, sensory perception disorders); (4) social adjustment and environmental factors (employment, family, sex, socialization and leisure time, legal problems); (5) substance use (alcohol, opiates, CNS depressants, CNS stimulants, hallucinogens, phencyclidine, cannabis, inhalants, polysubstance use); (6) substance use modalities (heroin intake, modality of use, stages of illness, nosography); (7) treatment history (previous and current treatments); (8) addiction history (age at first contact, age at onset of continuous use, dependence length, age at first treatment). Ten items are set up so as to elicit dichotomous answers (yes/no): (1) somatic comorbidities; (2) abnormal mental status; (3) work problems; (4) household problems; (5) sexual problems; (6) socialization and leisure time problems; (7) drug-related legal problems; (8) poly-substance abuse; (9) previous treatment; (10) combined treatments.

In the questionnaire, the modality of use is encoded according to Lahmeyer’s classification [41] as follows: (1) stables; (2) junkies; (3) two worlders; (4) loners. “Stables” are opioid addicts who have adopted conventional values, hold legitimate jobs, are generally law-abiding, and do not associate with other addicts. “Junkies” are closely identified with an addict subculture, are not legitimately employed, and subsist on the proceeds of criminal activities. “Two worlders” engage in criminal activities and associate with other addicts, but are legitimately employed. “Loners” are not involved neither in the addict subculture, nor the conventional culture: they are usually unemployed and live on welfare benefits, rather than on the proceeds of criminal activities.

The questionnaire does not provide statistic or psychometric indexes that require reliability or validity testing; it serves only as standardized medical record that we use in the routinely clinical practice.

### Toxicological urine analyses

Urine samples for toxicological analyses were randomly collected monthly to allow the evaluation of the presence of opiates, cocaine, cannabinoids, and their metabolites. The enzyme-multiplied immune technique was used for this purpose.

### Data analysis

We compared HRT and OAT patients for socio-demographic data, addiction history, urinalyses, and opioid medication doses. The variables that differed significantly between the two groups were included in separate logistic forward regression analyses, considering as a dependent variable whether one belonged to the OAT group or not. Statistical significance was set at *p* < 0.05. All *p* values were two-sided. Statistical analyses were carried out using the SPSS package 20.0.

## Results

### Socio-demographic features

In our study, 191 (77.3 %) patients were males and 56 (22.7 %) females. Patients’ mean age ± standard deviation (SD) was 33.64 ± 9.1 years (age range 16–59 years). Education length was less than 8 years in 169 (69.0 %) patients; 172 (70.8 %) patients were single; 85 (34.4 %) patients were unemployed. Economic condition was adequate in 205 (83.0 %) patients, whereas 23 (9.3 %) received welfare benefit. Forty-eight (19.4 %) patients lived alone.

Table 1 shows the main socio-demographic differences between HRT and OAT patients.

**Table 1:** Demographic and clinical differences between harm reduction treatment and opioid agonist treatment patients

	HRT strategy *N* = 189	OAT strategy *N* = 58	T/chi	*p*
Demographics				
Age, M ± sd	30.41 ± 6.3	44.14 ± 8.7	−11.09	0.000
Gender (females), *N* (%)	33 (17.5)	23 (39.7)	12.47	0.000
Education (low), *N* (%)	120 (63.5)	49 (87.5)	11.63	0.001
Single, *N* (%)	131 (70.1)	41 (73.2)	0.20	0.648
Unemployed, *N* (%)	66 (35.1)	19 (32.8)	4.77	0.189
Income (adequate), *N* (%)	151 (79.9)	54 (93.1)	5.48	0.019
Welfare benefit, *N* (%)	3 (1.6)	20 (34.5)	56.87	0.000
Living alone, *N* (%)	19 (10.1)	29 (50.0)	45.23	0.000
Clinical features at treatment entry				
Age heroin first use, M ± sd	18.64 ± 4.1	19.69 ± 5.1	−1.59	0.112
Age of continuous use, M ± sd	21.33 ± 4.5	23.02 ± 6.0	−1.96	0.053
Dependence length (years), M ± sd	7.30 ± 5.7	22.00 ± 9.1	−11.60	0.000
Age first treatment, M ± sd	24.72 ± 4.9	25.36 ± 6.3	−0.75	0.477
Somatic complications, presence, *N* (%)	130 (68.8)	51 (89.5)	9.64	0.002
Altered mental status, presence, *N* (%)	139 (73.5)	55 (98.2)	15.95	0.000
Job concerns, presence, *N* (%)	77 (41.6)	35 (66.0)	9.85	0.002
Household concerns, presence, *N* (%)	57 (30.5)	33 (57.9)	14.10	0.000
Loving concerns, presence, *N* (%)	64 (33.9)	38 (66.7)	19.41	0.000
Social-leisure concerns, presence, *N* (%)	74 (39.2)	33 (57.9)	6.25	0.012
Legal problems, presence, *N* (%)	56 (29.6)	36 (62.1)	19.98	0.000
Polyabuse, presence, *N* (%)	77 (40.7)	16 (28.6)	2.71	0.099
Past treated, *N* (%)	161 (85.2)	58 (100.0)	9.52	0.002
Combined treatments, *N* (%)	164 (86.8)	54 (94.7)	2.75	0.097
Heroin intake, daily or more, *N* (%)	113 (59.8)	41 (70.7)	2.24	0.134
Modality of use, unstable, *N* (%)	58 (30.7)	16 (28.1)	0.14	0.706
Periodic self detoxifications, *N* (%)	137 (72.5)	49 (84.5)	3.43	0.064
Stage 3 reached, *N* (%)	147 (77.8)	46 (79.3)	0.06	0.805
Dual diagnosis, presence, *N* (%)	108 (57.1)	32 (55.2)	0.07	0.791
Observational period (months), M ± sd	13.34 ± 3.0	18.68 ± 3.8	−9.69	0.000

Substitution medications	HRT strategy	OAT strategy	z*	*p*
	*N* = 102	*N* = 43		
Methadone dose, M ± sd	67.21 ± 25.8	53.12 ± 25.9	−2.65	0.008
	***N*** **=** **87**	***N*** **=** **15**		
Buprenorphine dose, M ± sd	7.29 ± 4.2	6.47 ± 4.4	−0.94	0.343

### Clinical features

OAT patients, more frequently than HRT ones, were affected by psychiatric and physical comorbidities and had been previously treated (Table 1). Their observation period was longer (Table 1). As regards addiction history, OAT patients showed a longer dependence duration than HRT ones. No difference was observed in terms of age of first heroin use, of continuous use, and of first treatment, as well as in terms of modalities of heroin use (Table 1).

### Urinalyses

Table 2 shows the mean and SD of clean urinalyses of HRT and OAT patients according to the substitution medication type. At the multivariate level, there was a statistically significant interaction between the effects of clean urinalyses and treatment modality (*p* = 0.002). Differently, the interaction between the effects of clean urinalyses and substitution medication type was not statistically significant (*p* = 0.118). The interaction between treatment modality and substitution medication type was not statistically significant (*p* = 0.512) (Table 2). Differences at the univariate level are reported in Table 2.

**Table 2:** Clean urinalyses percentage of harm reduction and agonist opioid treatment patients according to substitution medication used

Modality	Medication	Clean urinalyses		
		Heroin	Cocaine	Cannabinoid
HRT	Buprenorphine (*N* = 87)	0.88 ± 0.2	0.91 ± 0.2	0.75 ± 0.3
	Methadone (*N* = 102)	0.79 ± 0.3	0.86 ± 0.2	0.68 ± 0.3
	Total (*N* = 189)	0.83 ± 0.2	0.88 ± 0.2	0.71 ± 0.3
OAT	Buprenorphine (*N* = 15)	0.97 ± 0.1	0.97 ± 0.1	0.83 ± 0.3
	Methadone (*N* = 43)	0.93 ± 0.1	0.97 ± 0.1	0.70 ± 0.4
	Total (*N* = 58)	0.94 ± 0.1	0.97 ± 0.1	0.73 ± 0.4
Total	Buprenorphine (*N* = 102)	0.89 ± 0.2	0.92 ± 0.2	0.76 ± 0.3
	Methadone (*N* = 145)	0.83 ± 0.2	0.89 ± 0.2	0.68 ± 0.3
	Total (*N* = 247)	0.86 ± 0.2	0.90 ± 0.2	0.72 ± 0.3

Between-subjects effects				
Modality	Heroin urinalyses	*F* = 11.05; *df* = 1; *p* = 0.001		
	Cocaine urinalyses	*F* = 11.09; *df* = 1; *p* = 0.001		
	Cannabinoid urinalyses	*F* = 0.86; *df* = 1; *p* = 0.353		
Medication	Heroin urinalyses	*F* = 3.98; *df* = 1; *p* = 0.047		
	Cocaine urinalyses	*F* = 1.19; *df* = 1; *p* = 0.275		
	Cannabinoid urinalyses	*F* = 3.73; *df* = 1; *p* = 0.055		
Modality*medication	Heroin urinalyses	*F* = 0.68; *df* = 1; *p* = 0.409		
	Cocaine urinalyses	*F* = 1.13; *df* = 1; *p* = 0.289		
	Cannabinoid urinalyses	*F* = 0.37; *df* = 1; *p* = 0.542		

Multivariate test: modality effect: *F* = 5.24; *df* = 3; *p* = 0.002. Medication effect: *F* = 1.97; *df* = 3; *p* = 0.118. Modality*medication effect: *F* = 0.77; *df* = 3; *p* = 0.512

Table 3 shows that cocaine-clean urinalyses was the most discriminating characteristic of OAT patients (OR 7.47E+21).

**Table 3:** Most discriminant characteristics of OAT patients

Step	Predictors	*B*	Odds ratio	95 % CI	*p*
1	Dependence length	0.17	1.19	1.08–1.30	0.000
2	Duration of observation	0.38	1.46	1.17–1.82	0.001
3	Clean cocaine urinalyses	29.64	7.47E+12	46,674.13–1.20E+21	0.002

Statistic: Chi square 99.33, *df* 3, *p* = 0.000 correct classified 90.8 %

### Substitution medication

The mean methadone dose was lower in OAT than in HRT patients. No differences in buprenorphine doses were observed.

### Correlations between clean urinalyses and substitution medication doses

Regarding HRT patients, significant correlations were found between methadone dose and heroin-clean urinalyses (*p* = 0.017). High dosages correlated with a low percentage of heroin-clean urinalyses. A high percentage of heroin-clean urines correlated with a high percentage of cocaine- (*p* = 0.000) and cannabinoids- (*p* = 0.000) clean urinalyses. Considering OAT patients, no significant correlations were found between methadone or buprenorphine dose and clean urinalyses. A high percentage of heroin-clean urines correlated with a high percentage of cocaine-clean (*p* = 0.009) urinalyses.

## Discussion

Compared to HRT patients, OAT ones were older, more frequently females, more likely to live alone, and to have more workplace and justice issues. They had longer dependence duration, more severe addiction history, and received lower methadone doses. They were also observed for a significantly longer period. There was a higher proportion of heroin- and cocaine-clean urinalyses in OAT patients, with cocaine-clean urinalyses discriminating best between the two groups. Buprenorphine maintenance was more often associated with heroin-clean urinalyses. The higher the methadone doses, the lower were the percentage of heroin-clean urinalyses in HRT patients (negative correlation).

Our results are inconsistent with those of the Cochrane review by Faggiano et al. [23] that found that higher methadone doses (60–100 mg/day) were more effective than lower ones (1–39 mg/day) in reducing heroin use during methadone maintenance treatment. However, in line with our findings, several studies [10, 42, 43] failed to demonstrate a clear association between high methadone doses on the one hand and reduction of heroin use or clinical stabilization on the other hand. More specifically, Blaney and Craig [42] found no significant differences regarding any outcome variable (illicit drug use, treatment retention, missed medication days) by methadone dose and concluded that researchers should pay more attention to the interpersonal aspects of methadone maintenance treatment. Moreover, Reimer et al. [10], using the opiate dosage adequacy scale (ODAS) in a sample of patients in opioid replacement treatment, found that as many as 40.6 % suffered from an inadequate dosage, while 59.4 % had an adequate dosage. The “inadequate” group tended to receive higher doses of methadone when compared to the “adequate” group (70.6 vs. 57.8 mg). Trafton et al. [43] suggest that clinicians should individualize methadone doses as long as outcomes are satisfactory: extremely low doses (2 mg/day) may be effective in some patients, while extremely high doses (over 160 mg/day) are required in other patients to reduce or stop heroin use. Furthermore, in our HRT patients we found a positive correlation between methadone doses and the percentage of urines positive for heroin metabolites. We can hypothesize that the patients who received the highest methadone doses in clinical practice were those with a severe addiction, thus more difficult to treat. On the other hand, no significant correlation was found between methadone dosage and heroin use among OAT patients.

In our sample, about 10 % of urine toxicology tests were positive for cocaine, with significant differences between HRT and OAT patients. Similarly, other studies monitoring patients in opiate agonist treatment found cocaine-positive urines with toxicological values ranging between 4.3 and 13 % [10, 44].

Even though previous studies (Cochrane review, [23]) indicated that higher doses of methadone (60–100 mg/day) were more effective to reduce cocaine use in patients in opiate agonist treatment, we found no association between cocaine use and methadone dosage in both groups of patients.

Our findings are consistent with those of Epstein et al. [44], who compared patients randomly assigned to methadone doses of 70 and 100 mg/day, respectively. They found that the percentage of urines negative for cocaine or for opiates and cocaine simultaneously did not differ by dose. Noteworthy, Baumeister et al. [45] found that patients in the low dose group had significantly fewer cocaine consumption days than patients in the currently recommended dose range >60 mg/day. Moreover, in line with previous studies [45–47], we found a positive association between heroin and cocaine use in HRT and OAT patients. Our findings suggest that methadone maintenance has an important role in restraining most of the cases of pre-existing concurrent cocaine abuse. The onset of cocaine abuse during a successful methadone maintenance program is quite unlikely and is best predicted by former analogous abuse [48]. Finally, we found neither a significant difference in buprenorphine doses among OAT and HRT patients nor any significant associations between doses of buprenorphine and heroin or cocaine use in both groups of patients. Buprenorphine seemed to confirm its role in clearing heroin urinalyses better than methadone [49].

Regarding the percentage of cannabis use, no significant differences were found between HRT and OAT patients. Similarly to Epstein and Preston [50] we found that cannabis use was not associated with cocaine or heroin use.

In our opinion, it is of clinical interest that OAT patients, following a correct induction and once the stabilization phase has taken place, can obtain lower percentages of heroin and cocaine in urinalyses compared to HRT patients, even if treated with significantly lower doses of methadone. In addition, lower use of cocaine during OAT is the most discriminating characteristics of this kind of treatment when compared to HRT strategy. It must be underlined that, at treatment entry, OAT patients had a higher prevalence of physical and psychiatric comorbidities, a longer dependence history, greater work problems, more unsatisfactory households, and more legal problems than HRT patients.

Furthermore, our results support the hypothesis of Willembring et al. [51] about the stability of the clinic’s patient population: “Clinics with low turnover and a large number of patients that were stabilized on methadone years ago are likely to have lower average doses while still maintaining high concordance with dosing recommendations”.

In a paper published in 1965 [52], Dole and Nyswander observed that 22 heroin addicts had been stabilized by oral methadone. The medication seemed to have two useful effects: relief of narcotic hunger and induction of sufficient tolerance to block the euphoric effect of an average illegal dose of heroin. With this medication and a comprehensive program of rehabilitation the patients showed marked improvement, pointing to a good efficacy of the OAT strategy.

The limitations of this study are due to its retrospective design and to the small cohort of patients who underwent the assessment. A study designed specifically to elucidate this issue should be carried out. Assessments of the same patient in different clinical presentations of the natural history of heroin dependence would have provided more accurate information.

## Conclusions

Even patients with more severe substance use disorder and with multiple psychiatric, physical and social comorbidities, when treated with an appropriate long-term opioid agonist treatment and a multi-modal approach, could have a significant recover and reach satisfactory outcomes in term of substance abstinence.
