# Erratum to: Agonist Opioid Treatment as historical comprehensive treatment (‘Dole & Nyswander’ methodology) is associated with better toxicology outcome than Harm Reduction Treatment

**DOI:** 10.1186/s12991-017-0132-8

**Published:** 2017-02-13

**Authors:** Jacopo V. Bizzarri, Valentina Casetti, Livia Sanna, Angelo Giovanni Icro Maremmani, Luca Rovai, Silvia Bacciardi, Daria Piacentino, Andreas Conca, Icro Maremmani

**Affiliations:** 1 Department of Psychiatry of Bolzano, Bolzano, Italy; 2 Vincent P. Dole Dual Diagnosis Unit, Department of Neurosciences, Santa Chiara University Hospital, University of Pisa, Via Roma, 67 56100 Pisa, Italy; 3 AU-CNS-Association for the Application of Neuroscientific Knowledge to Social Aims, Pietrasanta, Italy; 4 G De Lisio Institute of Behavioural Sciences, Pisa, Italy

## Erratum to: Ann Gen Psychiatry (2016) 15:34 DOI 10.1186/s12991-016-0109-z

After publication of our article [1], we realised that the title used was incorrect. The correct title “Agonist Opioid Treatment as historical comprehensive treatment (‘Dole and Nyswander’ methodology) is associated with better toxicology outcome than Harm Reduction Treatment” appears above.

In addition, it has come to our attention that we did not declare a potential competing interest which is that Molteni Pharmaceutical agreed to provide funds to cover the article-processing charge before we submitted our article to *Annals of General Psychiatry*.
